# Detecting electrophysiological alterations in schizophrenia: an ERP-microstate analysis

**DOI:** 10.1192/j.eurpsy.2026.10635

**Published:** 2026-07-31

**Authors:** F. F. Marzocchi, A. Perrottelli, G. Di Lorenzo, C. Niolu, M. Altamura, A. Bellomo, P. Monteleone, P. Girardi, P. Bucci, A. Mucci, S. Galderisi

**Affiliations:** 1 Department of Psychiatry, University of Campania “Luigi Vanvitelli”, Naples; 2 Department of System Medicine, University of Rome “Tor Vergata”, Rome; 3 Department of Clinical and Experimental Medicine, University of Foggia, Foggia; 4 Department of Medicine, Surgery and Dentistry, ‘Scuola Medica Salernitana’, University of Salerno, Salerno; 5 Department of Neurosciences, Mental Health and Sensory Organs, University of Rome “La Sapienza”, Rome, Italy

## Abstract

**Introduction:**

Schizophrenia is a mental disorder whose pathophysiology is still not fully understood. Electroencephalographic (EEG) microstates (MS), which can be defined as instantaneous representations of scalp electrical activity, have been employed to investigate alterations in neural synchrony associated with this disorder. After being first characterized using resting-state EEG data, MS analysis has gained popularity as a useful tool for investigating spatial and temporal properties of event-related potentials (ERPs).

**Objectives:**

This study aims to investigate the alterations in parameters of MS in a sample of subjects with SCZ (SCZs), as compared to a group of healthy controls (HCs), and to explore their associations with negative symptoms and cognitive impairment.

**Methods:**

The analysis included EEG data from 132 SCZs and 65 HCs recorded during an auditory oddball task (320 standard tones, ST; and 80 target tones, TA). MS analysis was conducted using Cartool, and five parameters [Global explained variance (GEV), mean duration, time coverage, mean global field power (GFP), segment density] were extracted for each MS. Repeated-measures ANOVAs were performed to test group and group × condition interaction effects in each MS class. Correlation analyses of the extracted parameters with negative symptoms and cognitive impairment were performed.

**Results:**

Five MS maps (Image 1) were detected across the two study groups (SCZ and HC) and conditions (ST and TA). Visualization of the five maps showed that Map 1 had the typical topography of the N100 component (frontocentral negativity), while Map 2 was associated with the P3b component (parietal positivity).

The ANOVA showed a significant group effect for Map 1 and Map 2. For both maps, higher time coverage was observed in SCZ (p < 0.01), as compared to HC. Furthermore, GEV and mean duration of Map 3 (characterized by a central positivity) showed significant group and group x condition interaction effects (p < 0.01). Post hoc analysis showed lower GEV (only during the TA) and lower mean duration (during the ST and TA) in SCZ, as compared to HC (p < 0.01).

Correlation analyses showed that the expressive deficit domain of negative symptoms was negatively correlated (p < 0.05) with GEV of Map 1 in both ST (r = -0.22) and TA (r = -0.23) conditions and with mean duration of Map 1 during the TA condition (r = -0.27). Furthermore, significant positive correlations (p < 0.05) were observed between the attention-vigilance domain scores and GEV (r = 0.24), mean duration (r = 0.24) and time coverage (r = 0.23) of Map 3 during the ST condition within SCZ sample.

**Image 1:**
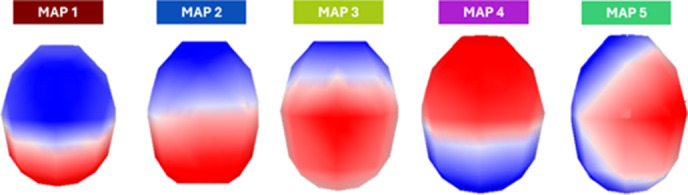

**Conclusions:**

The present study shows that MS analysis can highlight alterations in the temporal dynamics of scalp activity in subjects with schizophrenia during different stages of sensory and cognitive processing. Furthermore, specific parameters of MS are associated with psychopathological features and cognitive impairment.

**Disclosure of Interest:**

None Declared

